# Promoting Individual Learning for Trainees with Perceived High Helplessness: Experiences of a Safety Training Program

**Published:** 2014

**Authors:** Fariba Kiani, Mohamad Reza Khodabakhsh

**Affiliations:** 1 Student Research Committee, Ilam University of Medical Sciences, Ilam, Iran.; 2 Young Researchers & Elite Club, Mashhad Branch, Islamic Azad University, Mashhad, Iran.

**Keywords:** Attitude, Employees, Helplessness, Safety, Training

## Abstract

**Objective:**

The article arises from a research project investigating the effectiveness of safety training on changing attitudes toward safety issues. Followed by the training intervention was observed that employees’ helplessness decreased. The researchers have come to the idea of investigating how safety training can reduce perceived helplessness. Thus, this research examined the effectiveness of safety training on reducing employees’ helplessness with attention to the mediating role of attitude toward safety issues.

**Methods:**

The current study was an experimental study with the control group. A total of 204 (101 experimental group and 103 control group) completed safety attitude questionnaire and perceived helplessness before a safety training course including four 90-min sessions over 4 consecutive days in Esfahan Steel Company in 2012 between October and December. Only members of the experimental group participated in this course. These questionnaires, approximately 30 days later, again were run on members of both groups. Data were analyzed using descriptive indexes, t-, and F-test.

**Results:**

Results by comparing the two groups showed that safety training was effective only on individuals with perceived low helplessness (p = 0.02).

**Conclusion:**

In individuals with perceived high helplessness, safety training only with changing safety attitudes can reduce the perceived helplessness.

## Introduction

The steel industry has one of the highest incidents of fatal and non-fatal accidents/injuries every year. As a high risk industry, there is a need to investigate factors that affect the occurrence of these accidents to be able to protect workers (1). 

Organizing workers trainings and maintaining occupational safety and health in the workplace are vital for organizations and industries (2). The training is a process by which employees gain knowledge, learn new skills, or acquire motivation to start performing in a specific way (3). Providing enough training for all workers is crucial to ensure worker’s safety and to make them aware of any possible dangers and instruct ways to avoid occupational hazards (4). However, training is costly both in terms of production and expenses, so the evaluation of its effectiveness is necessary (5). The main emphasis on training effectiveness is making changes in safety performance (6). However, this might imply measuring accidents in the workplace as a basis for assessment of safety training, it show such problems like accidents being relatively low-frequent events and incidents have not being recorded in the workplace (7). One method with approved validity in relation to safety is the measurement of attitudes where they are specifically measured (8-11). Attitudes are related to occupational accidents and measuring them to evaluate the effectiveness of safety training can be useful (12). The safety attitudes are the beliefs and emotions around the safety issues, and they reflect a sense of responsibility and commitment toward safety issues (13, 14). Employees’ attitudes can act as a mediator between safety climate and accident occurrence and may indirectly influence the individuals’ safety behaviors and performance (13, 15). Researches show that safety attitude is related to other variables that associated with the occurrence of accidents such as: safety compliance practices (16), risk behavior (17), sensation seeking (18), breaking safety rules (19), and fatalism (14, 20).

Increasing attention in the literature focused upon learned helplessness (21). Helplessness related with coping style of denial and avoidance (22), decreasing the well-being (23), and increasing depression, anxiety and physical illness (24). It negatively related to work adjustment in newcomers (25) and positivity related to work alienation, include the deficiency in job involvement (26).

Individuals that feel they are exposed to uncontrollable job events and cannot do anything to change these events experience helplessness (27). Helplessness is a psychological state in which an individual to believe that no control over a circumstance environment that her action is meaningless, and it results from cognitive, motivational, and emotional deficiencies in individuals (28). Abramson et al. stated that the type of the individual’s attributions on uncontrollable events predict the degree and parameters of helplessness (29). An attribution is personal/stable/global predict serious consequences for a person, because the person (a) will search defect for the unpleasant events her/his inside, (b) believes that the outcome of events is stable, and (c) event will happen again (30). However, the mere observation of such deficiencies is not sufficient in order to conclude about the experience of helplessness, individuals should also perceive that the outcome of the event do not depend on their response (31).

With attention to stated material above, research about safety training and perceived helplessness can have many advantages for organizations and individuals due to increasing employees’ safe behaviors and promoting safety level in workplace. The present study attempts to: 1) the examination of the effectiveness of safety training to change the attitude toward safety issues; 2) the examination of the effectiveness of safety training to decrease perceived helplessness; 3) the specification of the mediator role of attitude toward safety issues in the relationship between safety training and perceived helplessness.

## Materials and Methods

***Participants***

The current study was an experimental study with the control group. This research was administrated between January and February 2012 in Esfahan Steel Company. Esfahan Steel Company (Zob Ahan-e Esfahan) opened in late 1960s, based close to the cities of Fooladshahr and Zarrinshahr, Esfahan Province. Esfahan Steel Company (ESCO) is the first and largest manufacturer of constructional steel products in Iran (No = 8300) (32). In this research, in attention to the extent and distribution of the employees in the different parts of Esfahan Steel Company (Tohid Building, Navard part, blast furnace, steel making, coke, fire, railway, gas, oxygen plant, technical guidance, etc.), the sample was selected according to the stratified random sampling method. In stratified random sampling, the strata are formed based on members’ shared attributes or characteristics. A random sample from each stratum is taken in a number proportional to the stratum’s size when compared with the population. These subsets of the strata are then pooled to form a random sample. Then simple random sampling or systematic sampling is applied within each stratum (33). The sample size was calculated using of SPSS for Windows (version 15, SPSS Inc., Chicago, IL, USA), Following the procedure recommended by Molavi (33). Given an, *α* level 0.05 and a power of 90%, the sample size required was estimated to be 204 subjects. Informed consent form was obtained from each participant and was approved the research by the appropriately constituted Ethics Committees at Isfahan University.

Participants ranged in age from 18 to 53; the mean age of the participants was 39 years (SD = 5.58 years); 62% of the participants were high school graduates, 38% were university graduates; 88% were married and 12% were unmarried; average work experience was 12 years (SD = 3.2 years) (Table 1).

**Table 1:** Demographic characteristics of the sample members (N = 204)

**Variables**	**Frequency **(%)
**Age (years)**	
**18-29 years**	36
**30-41 years**	36
**42-53 years**	28
**Sex**	
**Male**	90
**Female**	10
**Marital status**	
**Married**	88
**Single**	12
**Education**	
**University graduates**	38
**High school graduates**	62
**Work experience**	
**5 years and lower**	36
**6-15 years**	24
**16-25 years**	24
**26 years and higher**	16
**Shift ** **status**	
**Shift**	64
**Not shift**	36

Participants were randomly assigned into two groups: an experimental group (n = 101) and control group (n = 103). Using statistical methods: χ^2^ (gender), Student’s t-test (age and work experience), and Kruskal-Wallis H test (education level) was observed that there was no significant different between the experimental and control groups in terms of demography variables (p > 0.05). The experimental group received four 90-min sessions educational program over 4 days.

The trainees received safety training in four 90-min sessions over 4 consecutive days. In summary, topics of training session are presented in table 2.

The control group did not have training of any kind. All participants (both experimental and control groups) completed the questionnaires on safety attitudes and perceived helplessness pre- and 1-month later.

***Measures***

Validated instruments were used for data collection about attitude toward safety issues and perceived helplessness. At first, all questionnaires were translated from English into Persian and independently back-translated into English by a second translator. The few discrepancies between the original English and the back-translated version resulted in adjustment in the Persian translation based on direct discussion between the translators.

At the next step, psychometric characteristics of instruments were examined. Linguistic validation was performed by three experts of the psychology department and five experts of safety and health departments. Thus, the questionnaires were piloted and finalized with an advisory group of employees to ensure that the scales items were comprehensible and appropriate to the context. Moreover, conceptual analysis was confirmed the linguistic validity of all instrument. The questionnaires were distributed to employees with the help of union supervisor. Participants were assured of confidentiality, and informed consent in written format was acquired from each them.

**Table 2:** Headings of training session’ content

**Outline**
Welcome, Introduction and greeting
Express purposes of the meeting by some questions such as:
Which safety hazards threats a worker in his job?
Do the risks have long-term and short-term effects on employee’s health?
What can preventive measures do?
Safe working procedures in the job must be considered, what are these issues?
Notify participants with the vocabulary and safety reforms
Introduction to the kind of workplace and health risks that should be recognized
Identify risk areas
Familiar with personal protective equipment, how usage and benefits of their use
Description of how to properly work with machinery and equipments
Mention warnings on neglecting safety issues
Train emergency responses and how to provide first aid in crisis situations

**Table 3:** Components of attitude toward safety issues

Component	Explanation
Work conscientiousness	Refers to one’s sense of competence and responsibility.
Fatalism	Refers to views of importance and controllability of safety
Safety consciousness	Refers to one’s awareness of safety issues
Leadership	Refers to the satisfaction with the leadership (influence, inspirational motivation, intellectual simulation, individual consideration)
Role overload	Refers to perceptions about whether there is high workload in one’s job (i.e., too many hours worked per person)
Work pressure	Refers to work pace and availability of resources (i.e., time and workplace) available for the job
Job safety perception	Refers to a global perception over how safe one’s job is
Supervisor safety perception	Refers to perceptions about one’s supervisor behavior related to safety
Coworker safety perception	Refers to perceptions about one’s coworkers behavior related to safety
Management safety perception	Refers to perceptions about one’s company management attitudes and behaviors related to safety
Safety program and policies perception	Refers to perceptions about the safety program and polices in place
Interpersonal conflicts at work	Refers to the level respondents get along with others at work
Job involvement	Refers to beliefs regarding the importance the work plays in one’s life

Adapted for Munteanu (34), p. 22-23

*Attitude toward safety issues*

The safety attitude questionnaire used to collect data about attitude toward safety issues for this research was a self-reported questionnaire of Munteanu (34). This questionnaire is the method most often used for collecting attitudinal data and was therefore the choice for this research. This questionnaire translated and validated in Persian, and items of it were amended by safety and health specialists to adapt with the steel industry. The safety attitude inventory is a 66-item self-report scale that measure attitude factors related to accidents (Table 3).

It is presented in a multiple-choice format. The statements are arranged for reflect agreements’ intensity from strongly disagree (0) to strongly agree (4). A sample item is “I do not use equipment that I feel is unsafe”, that is related to safety consciousness factor. Munteanu (34) concluded that this inventory has high internal reliability (for all factors, Cronbach’s α takes values between 0.70 and 0.80) and also has a good validity. Evidence of reliability of this inventory, as administered to Iranian relevant populations, in this research, was calculated by α coefficient 0.78 and by Split-half 0.80 (for all factors, Cronbach’s α takes values between 0.56 and 0.87). The validity coefficients of questions and scales of safety attitude with other questionnaires of safety attitude are between 0.24 and 0.79 that all the validity coefficients are significant at p = 0.01.

*Perceived helplessness*

Perceived helplessness was measured with the 6-item of perceived helplessness scale developed by Cohen et al. (35). The items asked respondents how often they found their lives unpredictable, uncontrollable, and overloaded (36). All the items we used were modified to ensure that they were appropriate for the industrial context and were included a number of direct questions about the current levels of perceived helplessness. A sample item is “in the last month in the work environment, how often have you been angry because of the things that were outside of your control.” The questions in this scale ask about feelings and thoughts during the last month. In each case, respondents are asked about how often they felt in a certain way. Scoring is based on a Likert-scale format from never (0) to very often (4). The scores of participants were obtained by adding their responses to a 6-items questionnaire. Higher scores indicate that occupational events are perceived as unpredictable and uncontrollable by workers. This scale has validity (reliability = 0.84, 0.85, 0.86 in three cases), high internal reliability (0.79 = Cronbach’s α) and acceptable validity (37). Internal consistency (Cronbach’s α) in this study in Iran was 0.88, which was excellent for this scale. The validity coefficient of perceived helplessness scale with the attributional style questionnaire of Peterson et al. are 0.71 that this validity coefficient are significant at p = 0.01 (38).

***Data analysis***

From descriptive statistics and covariance analysis (in order to controlling for the effects of pre-test scores on post-test scores), were used to test the hypothesized relationships between safety training, perceived helplessness and attitudes toward safety issues, and this analysis was performed using software SPSS for Windows (version 18, SPSS Inc., Chicago, IL, USA). To test the mediating effect of attitudes toward safety issues on the relationship between safety training and perceived helplessness was used of equations designed by MacKinnon (39).

## Results

***Attitude toward safety issues***

We examined the data on attitude toward safety issues and perceived helplessness and found that there was not only homogeneity of variance, but in addition, the data were normally distributed. An ANCOVA analysis with removing the effect of pre-test scores indicated that there was a significant difference between the experimental group (M = 214.38, SD = 17.64) and control group (M = 202.03, SD = 15.2) on the measurement of attitude toward safety issues at the end of the training program [F(1, 202) = 8.12, p = 0.01]. Effect size was 0.15. The statistical power of 0.80 indicated that the sample size was sufficient to investigate this hypothesis.

***Perceived helplessness***

An ANCOVA analysis with removing the effect of pre-test scores indicated that there was a significant difference between the experimental group (M = 11.6, SD = 3.52) and control group (M = 14.31, SD = 4.75) on the measurement of perceived helplessness at the end of the training program (F(1, 202) = 14.22, p = 0.05). Effect size was 0.23. The statistical power of 0.96 indicated that the sample size was sufficient to investigate this hypothesis.

***Mediating effects***

To test the mediator role of attitude toward safety issues on the relationship between safety training and perceived helplessness, investigating correlation coefficients was observed that there is a significant overlap between the attitude toward safety issues and perceived helplessness (r = −0.31, p = 0.02); therefore, in order to investigate two possible alternative, was used of equations designed by MacKinnon (39), that is, the expansion of three-stage model proposed by Baron and Kenny (40) and Judd and Kenny (41).

*Attitude toward safety training mediated the effect of safety training on perceived safety helplessness*

The safety training had a significant positive effect on attitude toward safety issues [F(1, 202) = 14.22, p = 0.01] and perceived helplessness [F(1, 202) = 8.12, p = 0.05]. When perceived helplessness was regressed onto both attitude toward safety issues, and safety training, only safety training remained significant [F(1, 202) = 4.72, p = 0.03]. The attitude toward safety issues was no longer a significant predicator of perceived safety helplessness [F(1, 202) = 1.73, p = 0.13]. However, was observed that attitude toward safety issues has been somewhat mediated this relationship, so that the effect size is reduced (from 0.15 to 0.01). Contradictory results showed that, firstly, the attitude toward safety issues after arrival in this relationship as a third variable was not a significant predictor for perceived helplessness and also, its effect size was low (=0.04). Secondly, with the arrival of this variable as mediator, the effect size of safety training has somewhat decreased (p = 0.01). Therefore, maybe the overlap between safety attitudes and pre-test of perceived helplessness was caused to decrease the effect size of safety training. Thus, employees in terms of the mean score of perceived helplessness in the pre-test phase were divided into two groups. Individuals with low helplessness in group 1 and individuals with high helplessness in group 2 were placed, and the results of two groups were compared (Table 4).

As are shown in table 4, training safety was the significant predictor of perceived helplessness in the group with low perceived helplessness (p = 0.02), but in the group with high perceived helplessness, the attitude toward safety was the significant predictor of perceived helplessness (p = 0.02).

**Table 4:** The relationship between variables under study in individuals with perceived low and high helplessness

**Groups**	**Source of variation**	**Sum of squares**	**df**	**F**	**Sig**	**Effect size**	**Statistical power**
**Low helplessness**	Safety attitude	000.04	1	0.002	0.96	0.0005	0.050
	Group	105.15	1	6.020	0.02	0.2200	0.650
**High helplessness**	Safety attitude	075.62	1	6.090	0.02	0.2300	0.650
	Group	001.73	1	0.140	0.71	0.0060	0.065

**Figure 1:**
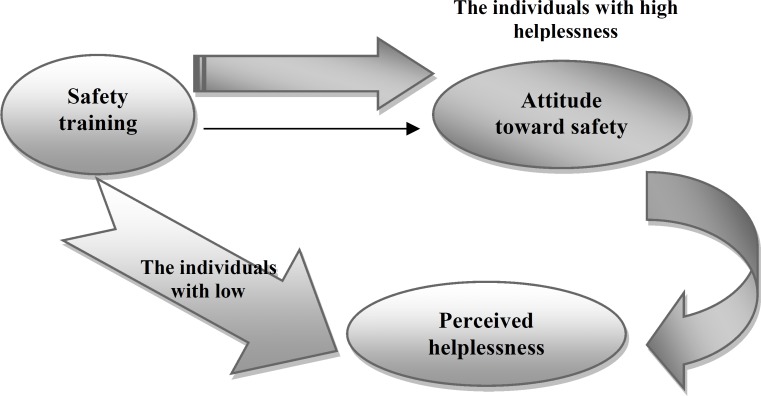
The relationships between research variables

*Perceived helplessness mediated the effect of safety training on attitude toward safety issues*

The safety training had a significant positive effect on perceived helplessness [F(1, 202) = 8.12, p = 0.05] and attitude toward safety issues [F(1, 202) = 14.22, p = 0.01]. When attitude toward safety issues was regressed onto both perceived helplessness and safety training, only safety training remained significant [F(1, 202) = 10.16, p = 0.02]. The perceived helplessness was no longer a significant predicator of attitude toward safety issues [F(1, 202) = 1.74, p = 0.51]. Also, was observed that the effect size was not considerably reduced (from 0.23 to 0.22). 

Therefore, the attitude toward safety issues mediated the relationship between safety training and perceived helplessness in the group with perceived high helplessness. In the group with perceived low helplessness, safety training decreased perceived helplessness. The relationships between these variables are presented in figure 1.

## Discussion

The current research examined the effectiveness of safety training on reducing employees’ helplessness with attention to the mediating role of attitude toward safety issues. 

The results of the present study indicated that safety training significantly improved employees’ attitude toward safety issues in the post-test. These results were aligned with findings of other research (42, 43, 12). Dong et al. showed that safety training play a positive role in improving occupational attitudes and consequently in reducing the number of job related accidents (43). Harvey et al. observed meaningful improvements in beliefs and attitudes before and after a safety training intervention (12). Colter (44) found that time of investigating an accident, important reasons that you hear from the employees, these are: “I did not know”, “I did not see”, “I did not think.” “I do not think I slip and fall”. He suggested that we must act proactively, only “training, training, training” is important. Quick et al. stated that the important predictor of the consideration of protective behaviors by individuals, are safety attitude (45). Safety training should have an effective role in improving safety attitudes and subsequently in promoting safety culture in organizations.

Furthermore, the current research results indicated that the safety training could improve perceived helplessness in post-test phase. For explaining the relationship, we can use of the uncontrollability content. The perception of helplessness usually occurs when a person has previously failed to achieve their career goals. If people think that they are unable to control events and attribute them to internal/stable/global causes, would perceive helplessness; Helpless individuals perceived future events uncontrollable (46).

With attention to in safety training courses, occupational accidents and how dealing with emergency situations be trained, this trainings can increase workers’ awareness about safety issues and reduce the individuals’ perception of uncontrollable events; subsequently decrease the perception of helplessness in employees. Individuals recognize the hazard conditions and realize that occupational accidents do not happen under any circumstances (doing a series of actions under certain conditions result to occurrence of these accidents). For example, with the consideration of safety issues and the use of protection devices decrease the injury chance of an individual in the heights. 

About the mediating effect of attitude toward safety issues on the relationship between safety training and perceived helplessness, the results showed that individuals with different levels of perceived helplessness in the pre-test safety were differently affected by the safety training. In the group with perceived low helplessness, safety training was the significant predictor of perceived helplessness and in the group with high helplessness, the attitudes toward safety issues was the significant predictor of perceived helplessness. This matter clarifies that individuals with low perceived helplessness have better attitudes toward safety issues; therefore, the feeling of their helplessness may relate to the lack of information and awareness about safety issues. Whereas, individuals with high perceived helplessness have weaker attitude toward safety issues. Providing safety trainings alone aren’t adequate for them; only training that can change attitudes toward safety issues, can be effective in reducing their helplessness.

According to these results, we can say that one of the causes of perceived helplessness in employees can be employees’ weak attitudes toward safety issues and can decrease the perceived helplessness with promoting their safety attitudes. This result can be justified with two constructs of fatalism and locus of control.

Fatalism is an obstacle to the adoption of safe working behavior (47). Fatalism describes the belief that injuries are unavoidable and happen due to haphazard or fate (20). It is negatively related with reporting job risk (48) and is positively related with the self-care disorder (49). Believe to fatalism have negatively influenced the acceptance of safe work practices (50). Fatalism can be recognized by perceptions of worthlessness, powerlessness, hopelessness, and futile (51). Believe to fatalism may facilitate the attitude that accidents are unprofitable (52), and consequently, increases helplessness in among workers. The results of Patwary et al. showed that fatalistic beliefs among personnel of an organization that attributed these events to “*fate*” reflecting their perceived lack of control over accidents and reveals a lack of organizational awareness that can occur within a culture of fatalism (53). Safety training with increasing employees’ awareness and with changing their attitudes toward safety issues can change this culture in organizations, and can improve employees’ control on safety issues. 

External locus of control refers to attribute consequence to external element. In contrast internal locus of control refers to attribute consequence to internal element (54). Employee with an internal locus of control tends to believe that they can prevent accidents and injuries. In contrast, employee with an external locus of control tends to believe that accidents and injuries are due to forces outside their control, such as fate, or fatalism (55). External locus of control has a positive correlation with helplessness (56) and accident occurrence (57), and internal locus of control has a negative correlation with psychological distress (58), anxiety and depression (59). Individuals with an internal locus of control have better attitudes toward safety issues, thus the perception of helplessness in them more likely returns to lack of awareness. In contrast Individuals with an external locus of control have weaker attitudes toward safety issues (60), to decrease helplessness in them, should early be promote their attitudes toward safety issues. 

The present study needs to be replicated in different populations and needs more empirical support. Until then, the findings of the study should be interpreted with caution. Further, the cross-sectional design of the study and participants (i.e., a group of employee) exert some limitations on the generalization of the findings. Finally, the problems and limitations on the use of self-repotting instruments should not be overlooked. However, limitation is usually accepted due to the fact that self-report surveys are considered the most practical way to collect data and to reflect individual attitudes and behaviors. There are many aspects which should be clarified in future research, for example, do personality styles of worker have mediating role in relation between safety training and perceived helplessness? These observations would be investigated in the future research. 

## Notes

**Declaration of interest:** None.
